# High-intensity high-volume swimming induces more robust signaling through PGC-1α and AMPK activation than sprint interval swimming in *m*. *triceps brachii*

**DOI:** 10.1371/journal.pone.0185494

**Published:** 2017-10-03

**Authors:** Rafael A. Casuso, Julio Plaza-Díaz, Francisco J. Ruiz-Ojeda, Jerónimo Aragón-Vela, Cándido Robles-Sanchez, Nikolai B. Nordsborg, Marina Hebberecht, Luis M. Salmeron, Jesus R. Huertas

**Affiliations:** 1 Department of Physiology, School of Pharmacy, University of Granada, Granada, Spain; 2 Institute of Nutrition and Food Technology “José Mataix,” Biomedical Research Center, University of Granada, Granada, Spain; 3 Department of Biochemistry and Molecular Biology II, School of Pharmacy, University of Granada, Granada, Spain; 4 Department of Nutrition, Exercise and Sports, Section of Human Physiology, University of Copenhagen, Copenhagen, Denmark; 5 San Cecilio University Hospital, Granada, Spain; Universidad Europea de Madrid, SPAIN

## Abstract

We aimed to test whether high-intensity high-volume training (HIHVT) swimming would induce more robust signaling than sprint interval training (SIT) swimming within the *m*. *triceps brachii* due to lower metabolic and oxidation. Nine well-trained swimmers performed the two training procedures on separate randomized days. Muscle biopsies from *m*. *triceps brachii* and blood samples were collected at three different time points: a) before the intervention (pre), b) immediately after the swimming procedures (post) and c) after 3 h of rest (3 h). Hydroperoxides, creatine kinase (CK), and lactate dehydrogenase (LDH) were quantified from blood samples, and peroxisome proliferator-activated receptor γ coactivator 1α (PGC-1α) and the AMPK^pTHR172^/AMPK ratio were quantified by Western blot analysis. PGC-1α, sirtuin 3 (SIRT3), superoxide-dismutase 2 (SOD2), and vascular endothelial growth factor (VEGF) mRNA levels were also quantified. SIT induced a higher release of LDH (*p* < 0.01 at all time points) and CK (*p* < 0.01 at post) than HIHVT, but neither SIT nor HIHVT altered systemic hydroperoxides. Additionally, neither SIRT3 nor SOD2 mRNA levels increased, while PGC-1α transcription increased at 3 h after SIT (*p* < 0.01) and after HIHVT (*p* < 0.001). However, PGC-1α protein was higher after HIHVT than after SIT (*p* < 0.05). Moreover, the AMPK^pTHR172^/AMPK ratio increased at post after SIT (*p* < 0.05), whereas this effect was delayed after HIHVT as it increased after 3 h (*p* < 0.05). In addition, VEGF transcription was higher in response to HIHVT (p < 0.05). In conclusion, SIT induces higher muscular stress than HIHVT without increasing systemic oxidation. In addition, HIHVT may induce more robust oxidative adaptations through PGC-1α and AMPK.

## Introduction

It is well-established that sprint interval training (SIT), conducted as cycling or running, induces mitochondrial biogenesis in human *m*. *vastus lateralis* [1,2]. However, recent findings suggest that two weeks of SIT arm cycling apparently fails to do so in the *m*. *triceps brachii* of untrained subjects despite marked elevations of arm-cranking VO_2peak_ and work capacity [3]. It is possible that *m*. *triceps brachii* maladaptability exists as a result of excessive generation of mitochondrial reactive oxygen species (ROS) specific to the muscle group as indicated by the marked increase of catalase, which elevates the capacity for H_2_O_2_ decomposition as well as a reduction of the redox sensitive tricarboxylic acid cycle enzyme aconitase, resulting in inhibited maximal mitochondrial respiration [3]. However, a recent study suggest that the *m*. *deltoideus* mitochondrial content can be increased in untrained women after 12 weeks of high-intensity swimming [4], which is in apparent contrast to the *m*. *triceps brachii* observations.

Front crawl swimming primarily involves the posterior part of *m*. *deltoideus* as well as *m*. *triceps brachii* [5]. Thus, trained swimmers appear to be the optimal model to investigate whether mitochondrial-related molecular signaling can be induced in *m*. *triceps brachii*. Furthermore, analysis of the response to both high-intensity high-volume training (HIHVT) swimming and SIT swimming exercise provides the possibility to evaluate the effect on markers of muscular stress, systemic increased oxidation, and the associated molecular signaling. Muscular stress and disruption can be monitored by evaluating circulating levels of creatine kinase (CK) and lactate dehydrogenase (LDH). Moreover, systemic oxidative stress can be monitored by determining plasma hydroperoxides (HPx) [6,7].

Peroxisome proliferator-activated receptor gamma coactivator 1-alpha (PGC-1α) is a key regulator of oxidative-related adaptations to exercise through controlling mitochondrial biogenesis [8,9] and the angiogenic response [10]. In addition, PGC-1α regulates the transcription of antioxidant enzymes, thus providing cellular protection against excessive oxidative stress [12,13]. Specifically, an association between the expression of PGC-1α and superoxide-dismutase 2 (SOD2) has been described in a variety of tissues [11,12,14], in a mechanism likely regulated by sirtuin 3 (SIRT3) [15–17]. Recent findings in rodents suggest that PGC-1α controls the antioxidant effects of exercise through a mechanism mediated by both SOD2 and SIRT3 [18]. In humans, however, the relationship between SIRT3 and PGC-1α in response to exercise is still unclear [19].

PGC-1α is regulated in response to acute exercise by several mechanisms. The high NAD^+^ levels generated within contracting cells activate the NAD+ protein deacetylase SIRT1 [20]. SIRT1 induces the transcription of PGC-1α through a mechanism likely mediated by AMPK [20,21]. Moreover, both proteins regulate the activity of PGC-1α [22]. Therefore, this study investigated the hypothesis that SIT compared to HIHVT is associated with increased oxidation and reduced signaling of AMPK and PGC-1α in *m*. *triceps brachii*.

## Methods

### Subjects

Nine male swimmers enrolled in swimming competitions for at least 8 years (y) and in swimming training for at least 13 y volunteered for the study. Their physical characteristics were as follows: age 23.0 y (range 19–26 y), weight 78.5 kg (range 63–92 kg), height 180 cm (range 174–187 cm), extended upper limb size 187 cm (range 180–197 cm), and a maximal swimming speed of 2.0 m/s (range 1.88–2.10 m/s) (Table 1). Maximal swimming speed was recorded several times before (~1 wk) the initiation of the study, and an experienced swimming coach, independent of the study, recorded the time required for the 25 m sprint, pushing off from the wall in the swimming pool. All subjects were fully informed of the risks and discomfort associated with the study, and all signed written consent. The study was conducted in accordance with the guidelines contained in the Declaration of Helsinki II and was approved by the local ethics committee of the University of Granada, Spain (protocol: 23/CEIH72015).

**Table 1:** Subject characteristics.

	Age (y)	Height (m)	Weight (kg)	Extended upper limb size (m)	Maximal velocity (m/s)
Subject 1	26	1.74	80	1.76	1.95
Subject 2	24	1.81	90	1.95	2.10
Subject 3	21	1.75	75	1.82	2.05
Subject 4	19	1.81	74	1.80	1.88
Subject 5	25	1.85	92	1.89	2.00
Subject 7	20	1.76	63	1.80	1.91
Subject 8	24	1.84	77	1.94	2.01
Subject 9	24	1.87	80	1.97	2.07

Maximal swimming velocity was calculated from a maximal 25m sprint pushing off from the wall of the swimming pool. y, years; m, meters; kg, kilograms; s, seconds.

### Experimental design

We used a crossover study design in which all subjects attended three experimental days. The first experimental day consisted of recording anthropometric measurements and the collection of basal muscle biopsies and blood samples at the Department of Physiology and Angiogenesis at Virgen de las Nieves Hospital (Granada, Spain). During the second and third days, the subjects performed two different swimming protocols differing in the intensity and volume applied each day. The second and third experimental days were performed at the 25 m indoor swimming pool of the Faculty of Sports, University of Granada, Spain. All the interventions were conducted within 14 days and swimmers were asked to refrain from physical activity 48 h before each experimental day. The experimental interventions were separated at least by 7 days. Before the first experimental day and including a light breakfast, subjects recorded their three-day food intake and they were asked to repeat this eating pattern before each experimental day. During the second and third experimental days, the subjects performed two swimming sessions; one consisted of ten bouts of 200 m at high steady-state intensity and the other protocol chosen was ten bouts of 50 m all- out sprint (see *Swimming interventions*). The swimming session on the first day was chosen randomly and on the second day the swimmers performed the other swimming protocol. Two experienced coaches, independent of the study objectives, recorded the time spent within each bout and monitored heart rate (HR) during each recovery period while the swimmers rested in the swimming pool. Once the swimming protocols had been completed, muscle and blood samples were obtained from the *m*. *triceps brachii* post exercise and after 3 h of rest.

All biopsies were taken between 10:00 am and 14:00 pm. During the entire protocol, including the 3 h post-exercise recovery, swimmers were asked to drink water *ad libitum*, but no other food or liquid was allowed.

### Swimming interventions

Once in the swimming pool, participants completed a standardized warm-up consisting of 5 min of articular mobilization, 400 m easy swimming, 5 min of leg-only swimming and 5 min of arm-only swimming, 5 min of aquatic skills, and four 25 m sets of progressive intensity swimming [23]. The SIT protocol consisted of ten bouts of 50 m all-out effort every 4 min; the swimmers were asked to perform each bout as fast as possible and were continuously encouraged to perform the best time in each bout. The HIHVT protocol was a modification of the 2000 m test protocol described by Maglischo [24]. The swimmers performed 10 bouts of 200 m interspersed by 40 s during which they were asked to maintain a higher average swimming speed throughout the total 2000 m. Between each bout, the swimmers rested passively inside the swimming pool. All swimming bouts were initiated within the swimming pool by pushing off from the wall. A 6–20 rating of the perceived exertion (RPE) scale was recorded immediately after the cessation of each swimming bout.

### Heart rate, blood lactate concentration, and blood collection

Upon arrival at the swimming pool, the swimmers rested, lying in a supine position for 10 min and the basal blood lactate concentration was determined (Lactate Pro, Japan) using a 5 μL fingertip blood sample. Immediately after each 50 m or 200 m bout, HR was monitored using a Polar Team 2 (Finland). Maximal HR was considered to be the maximal level recorded during the first 10 s after cessation of the bout. When the HR took more than 10 s to be monitored, probably due to water and/or chlorine, the measurement was not used for the calculations. Upon cessation of the protocol, blood lactate was measured at 3, 7, and 15 min post exercise.

Blood samples (5 mL) were collected from the antecubital vein on the basal day (pre), 3 min after swimming (post) and after 3 h of rest (3 h). Blood samples were collected into BD Vacutainer^®^ tubes (Becton Dickinson, NJ, USA). An aliquot of the blood centrifuged for 10 min at 1000 x g and 4°C to separate serum and plasma, both of which were stored at -80°C until further analysis.

### Systemic lipid peroxidation and muscle damage quantification

Plasma HPx were determined by spectrophotometry using the Sigma PD1 kit (St Louis, MO, USA) and following the manufacturer’s recommendations. This method has been described previously [7]. Absorbance changes at 560 nm were monitored spectrophotometrically.

Serum myocardial isoform (MB) of CK and LDH were quantified as markers of muscle damage. These were measured by spectrophotometry, according to the manufacturer’s recommendations, by a manual procedure using commercial kits (Spinreact, S.A. Girona, Spain, for LDH, and Spinreact, S.A. Girona, Spain, for CK-MB). HPx, CK, and LDH data were normalized to pre levels. These markers were included to evaluate the muscular damage and oxidative stress effects imposed by the two different exercise intensities under the hypothesis that HIHVT would not result in severe oxidative stress and thus could possibly be more beneficial than SIT for inducing skeletal muscle adaptations.

### Muscle biopsies

All skeletal muscle tissues were collected from the long head of the *m*. *triceps brachii*. The procedure was performed while the subjects were lying face down. The skin, subcutaneous tissue, and fascia were anesthetized by injecting lidocaine at 2%. A muscle sample of ~20 mg was taken (Coaxial Achieve 16G x 11 cm, ref.CA1611), and visible fat and blood were removed. Post-exercise muscle biopsies (post) were taken 5–7 min post exercise. Afterwards, at 3 h post exercise, the procedure was repeated ~2 cm apart from the post-exercise biopsy area. The samples were immediately frozen in liquid nitrogen and stored at -80°C until subsequent analysis. Thus, to investigate the acute effect of exercise at different intensities on mitochondrial biogenesis and the regulation of antioxidant enzyme expression, transcripts related to the PGC-1α pathway were quantified in *m*. *triceps brachii*.

### Quantitative real time (qRT)-PCR

We used the Real-time Ready Custom Panel 96 (Roche, Barcelona, Spain), which is a two-step qRT-PCR platform. Briefly, total ribonucleic acid (RNA) was extracted from the *m*. *triceps brachii* using the PeqGOLD HP Total RNA kit (Peqlab, Germany), according to the manufacturer’s recommendations. Isolated RNA was then treated with Turbo DNase (Ambion, Life Technologies, Carlsbad, CA, USA). The final RNA concentration and quality were determined using a NanoDrop2000 (NanoDrop Technologies, Winooski, Vermont, USA). Complementary DNA (cDNA) was synthesized from total RNA using the iScript advanced cDNA Synthesis Kit (Bio-Rad Laboratories, California, USA). The RealTime Ready Custom Panel 96 (Roche, Barcelona, Spain) included the following specific primer pairs: PGC-1α (Assay ID 101605, Roche, Barcelona, Spain), SIRT1 (Assay ID 102150, Roche, Barcelona, Spain), SIRT3 (Assay ID 140718, Roche, Barcelona, Spain), SOD2 (Assay ID 111256, Roche, Barcelona, Spain), VEGFA (Assay ID 140396, Roche, Barcelona, Spain), PTGS1 (COX-1) (Assay ID 111251, Roche, Barcelona, Spain), NRF1 (Assay ID 119207, Roche, Barcelona, Spain), and TFAM (Assay ID 115003, Roche, Barcelona, Spain). The HPRT-1 (Assay ID 102079, Roche, Barcelona, Spain), HMBS (Assay ID 102110, Roche, Barcelona, Spain), and ACTB (Assay ID 143636, Roche, Barcelona, Spain) genes were used as reference genes. The cDNA was then subjected to qRT-PCR with the LightCycler^®^ 480 Probes Master Kit (Roche, Barcelona, Spain) on a LightCycler^®^ 480 Instrument II detector (Roche, Barcelona, Spain). The PCR conditions were 1 cycle of 95°C for 10 min, followed by 45 cycles of 95°C for 10 s, 60°C for 30 s, and 72°C for 1 s, and 1 cycle of 40°C for 30 s. The expression level of each gene was analyzed with RT^2^ Profiler PCR Array Data Analysis software (version 3.4, SABiosciences). Changes in gene expression were expressed as fold changes (Fc).

### Western blotting

The *m*. *triceps brachii* samples were harvested in 10 mM Tris-HCl (pH 7.5), 150 mM NaCl, 2 mM EDTA, 1% Triton X-100, 10% glycerol, and a protease inhibitor cocktail (Thermo Scientific), and were placed on ice for 20 min. After centrifugation (30 min, 13,000 g, 4°C), the protein content in the supernatant was determined using the Protein Assay Kit II (Bio-Rad Laboratories, California, USA). Samples containing 30 μg of protein were mixed with 3X SDS-PAGE sample buffer (100 mM Tris-HCl, pH 6.8, 25% SDS, 0.4% bromophenol blue, 10% β-mercaptoethanol, and 2% glycerol), separated via SDS-PAGE using TGX Any kD gel (Bio-Rad Laboratories, California, USA), and transferred onto a nitrocellulose membrane (Bio-Rad Laboratories, California, USA). After incubation in blocking buffer (5% non-fat milk and 1% Tween 20 in Tris-buffered saline, TBS), the membranes were probed with the following antibodies: rabbit anti-total-AMPKα (D5A2) and rabbit anti-phosphorylated-AMPKα (40H9, phospho-AMPKα-Thr-172) (both 1:1000 in 5% BSA), purchased from Cell Signaling Technologies (Beverly, MA, USA); rabbit anti-PGC-1α (H-300) (dilution 1:500 in 5% non-fat milk) antibody, acquired from Santa Cruz Biotechnology (Santa Cruz, CA, USA); mouse anti-α-tubulin (internal control, 1:4000 in 5% non-fat milk), obtained from Sigma (Sigma-Aldrich, St. Louis, MO, USA). Immunoreactive signals were detected via enhanced chemiluminescence (SuperSignal West Dura Chemiluminescent Substrate, 34075, Thermo Scientific, Europe), and the membranes were digitally imaged and quantified through densitometry using ImageJ software. The results are represented as the Fc in expression relative to the control. The graph shows a representative crop blot.

### Statistical analysis

Data are represented as the mean ± standard error of the mean (SEM). The normal distribution of variables was tested with the Kolmogorov–Smirnov test. Analysis of covariance (ANCOVA) adjusted for age and maximal velocity was used to analyze changes in performance, HR, blood lactate, and RPE. Paired t-test was used to determined differences in normalized CK, LDH, and HPx levels. Significant differences in gene expression and protein levels were determined using the non-parametric Mann-Whitney *U* test; statistical significance was defined as *p* < 0.05. Statistical analyses were performed using SPSS version 22, for Windows (SPSS, Chicago, IL, USA).

## Results

### Swimming intensity and physiological response

**Fig 1:**
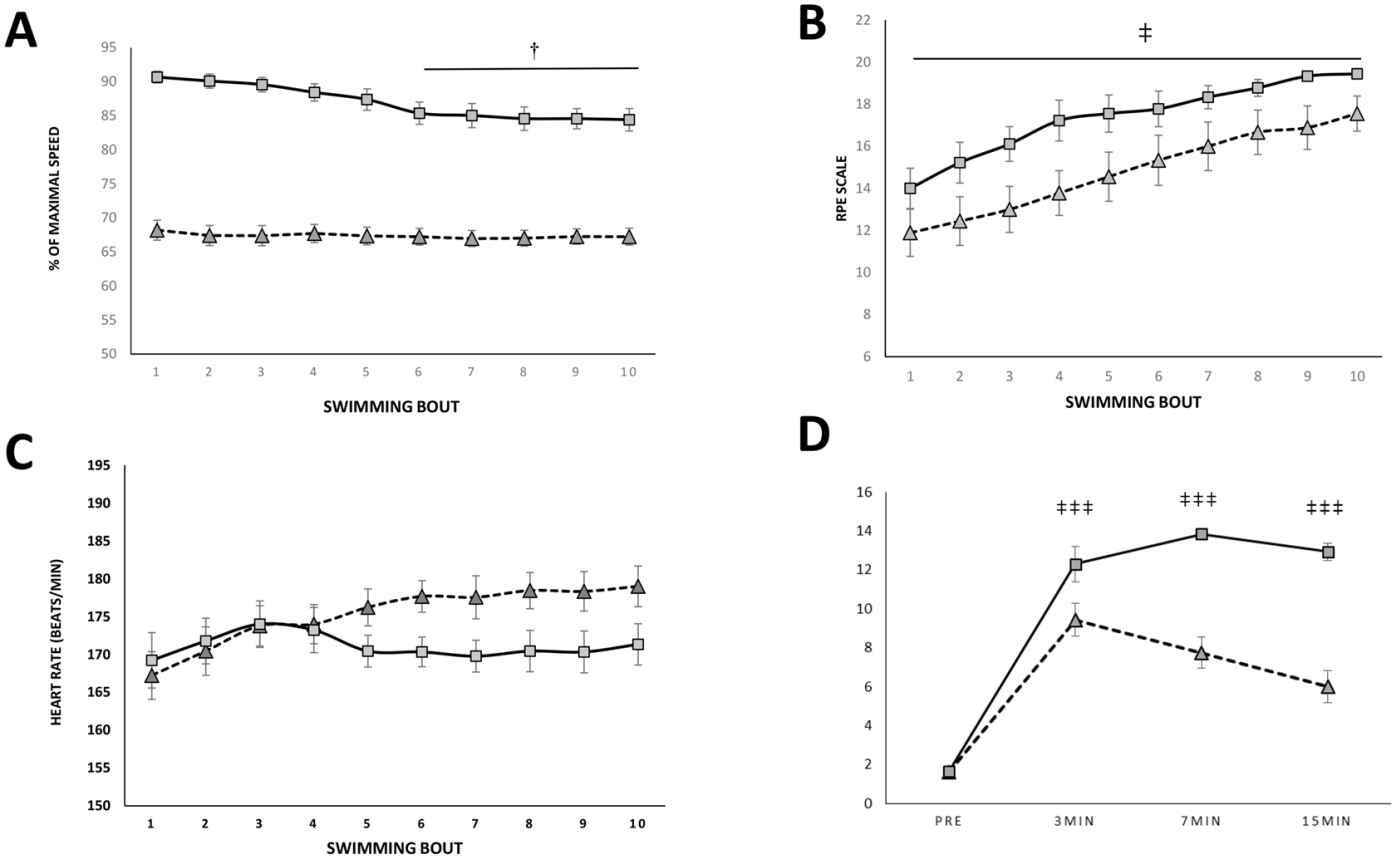
Swimming protocols differ in blood lactate production and perceived exertion but not in heart rate. Swimming performance, in terms of percentage of maximal speed, decreased in response to SIT (squares) after the sixth bout, while it remained stable during the HIHVT protocol (triangles) (A). Rate of perceived exertion was higher for SIT compared to HIHVT after every bout (B). No significant differences were found in heart rate (C). Blood lactate was higher after SIT at each of the time points examined (D). Results are shown as mean ± SEM. † *p* < 0.05 significantly different from the first swimming bout. ‡ *p* < 0.05, ‡‡‡ *p* < 0.001 significantly different from HIHVT.

Swimmers performed the SIT protocol at an average pace of 28.8±1.4 s over 50 m, which represents 86.8±3.9% of the maximal swimming speed. The HIHVT protocol was completed at 67.3±3.8% of maximal speed, with an average pace of 149.0±8.2 s over 200 m. The participants maintained a constant speed within HIHVT (Fig 1A). In contrast, there was a loss of performance after the sixth bout in the SIT protocol (Fig 1A). No differences were found for HR between SIT and HIHVT (Fig 1B), but RPE was higher (*p* < 0.05) in response to SIT throughout the protocol (Fig 1C). For blood lactate (Fig 1D), SIT induced higher concentrations than HIHVT at every time point (*p* < 0.001).

### Membrane damage and systemic oxidative stress

**Fig 2:**
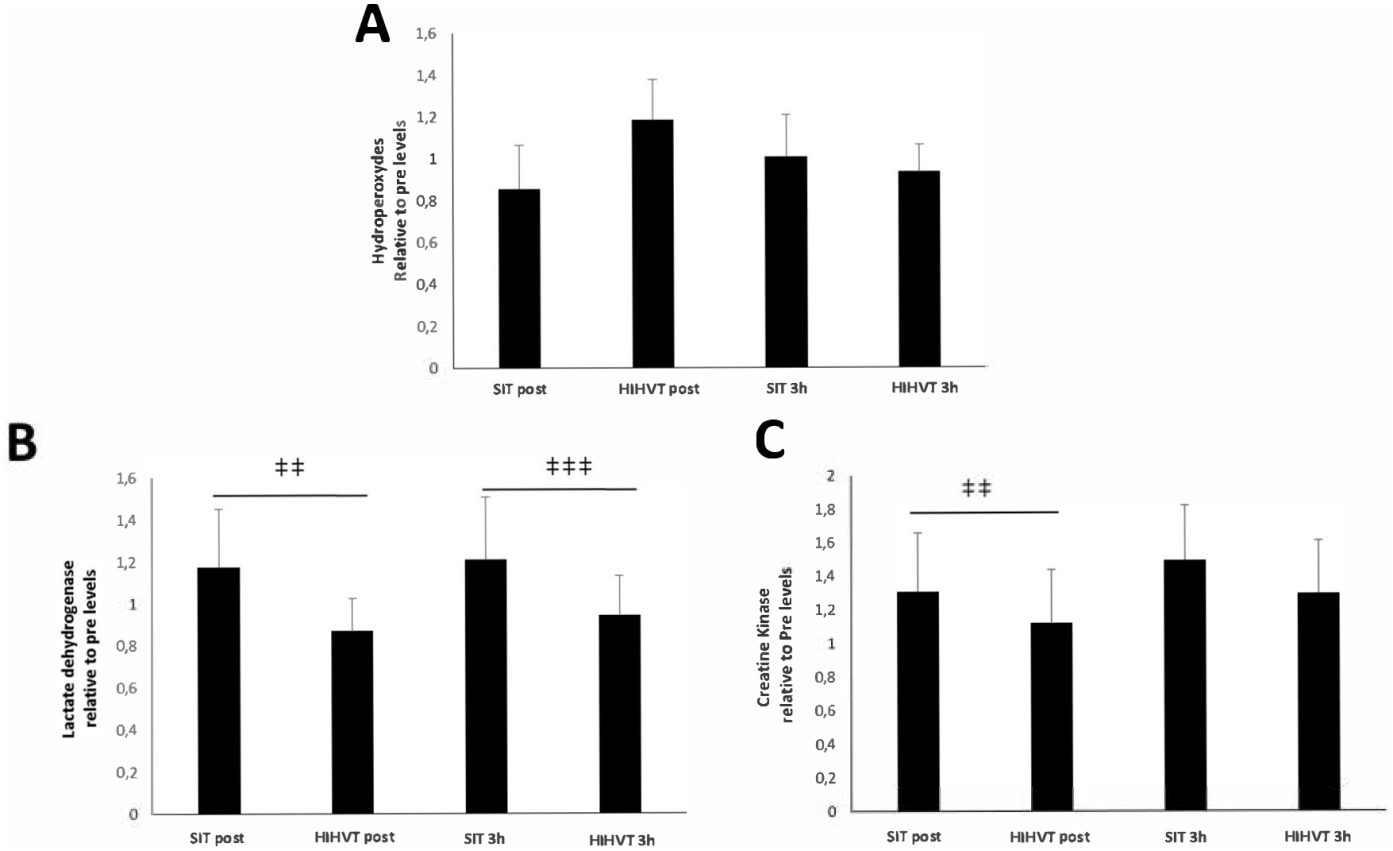
Lipid peroxidation and muscle damage. Circulating hydroperoxides were unchanged in response to both HIHVT and SIT (A). Lactate dehydrogenase was higher after SIT both at post and at 3 h (B). Creatine kinase was higher after SIT at post (C). Results are shown as mean ± SEM. ‡‡ *p* < 0.01, ‡‡‡ *p* < 0.001 *vs*. HIHVT.

Lipid peroxidation, quantified as systemic HPx levels, was similar for SIT and HIHVT (Fig 2A). Nevertheless, SIT induced higher LDH levels (Fig 2B) at post (*p* < 0.01) and at 3 h (*p* < 0.001). SIT also induced higher CK release (Fig 2C) than HIT at post (*p* < 0.01) but not at 3 h.

### Muscle signaling

**Fig 3:**
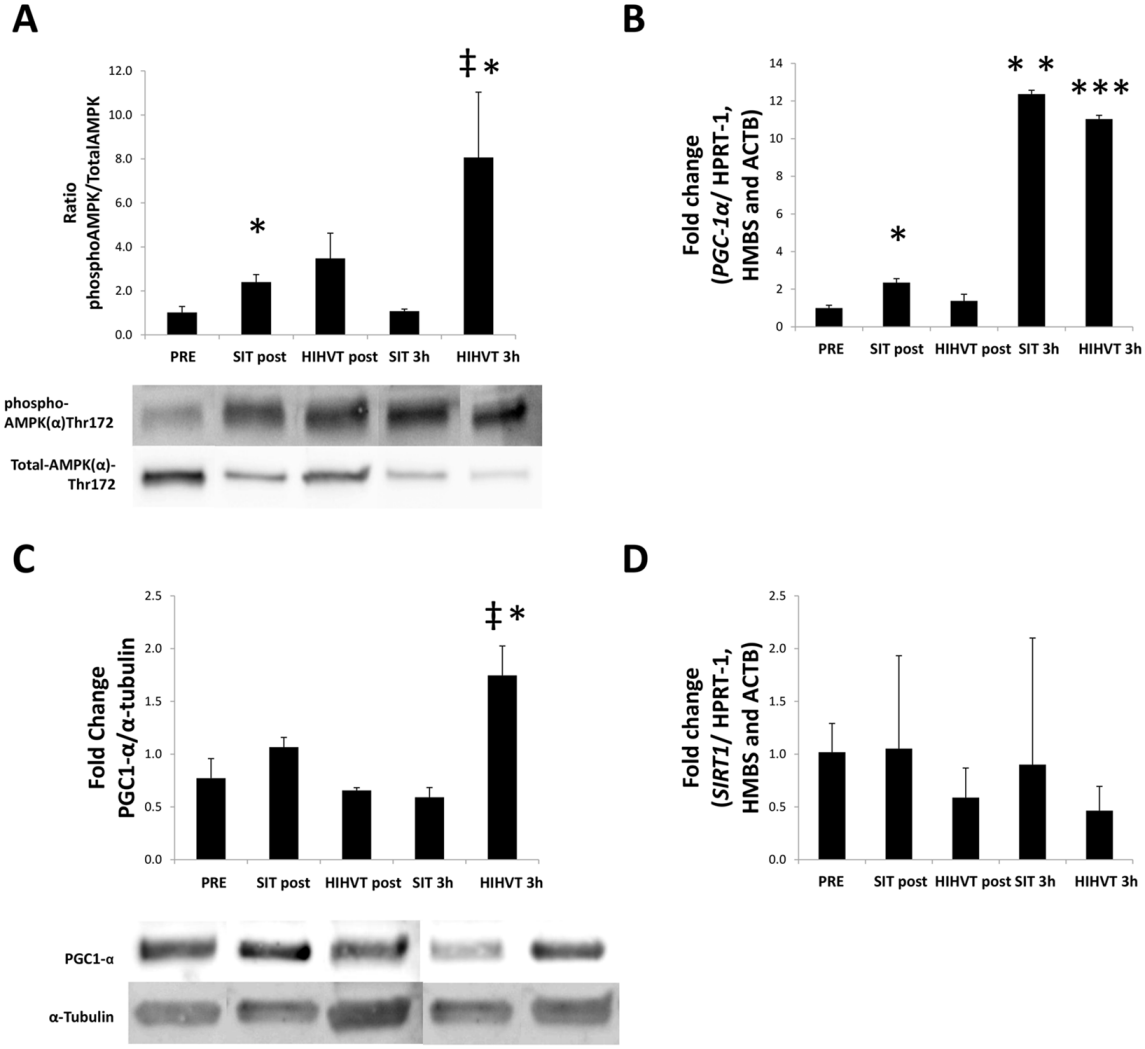
Molecular signaling in response to swimming. The ratio of AMPK phosphorylation at Thr172/total AMPK increased at post in response to SIT and at 3h in response to HIHVT (A). The PGC-1α mRNA levels increased at post in response to SIT and at 3 h in response to both SIT and HIHVT (B). Content of PGC-1α protein increased in response to HIHVT at 3 h (C). SIRT1 remains unchanged throughout all the time points examined (D). Results are shown as mean ± SEM. The graph shows a representative crop blot. * *p* < 0.05 ** *p* < 0.01 *** *p* < 0.001 *vs*. pre. ‡ *p* < 0.05 *vs*. SIT.

With regard to molecular signaling, the AMPK^pTHR172^/AMPK ratio increased significantly in response to SIT at post in *m*. *triceps brachii* (Fig 3A) (*p* < 0.05). The AMPK^pTHR172^/AMPK ratio was also higher in response to HIHVT but at 3 h (*p* < 0.05). Importantly, AMPK^pTHR172^/AMPK ratio was higher for HIHVT than for SIT at 3 h (*p* < 0.05). The PGC-1α mRNA levels were up-regulated in response to SIT at post (*p* < 0.01) (Fig 3b) and markedly elevated at 3 h after both SIT (*p* < 0.01) and HIHVT (*p* < 0.001). However, PGC-1α protein levels increased only in response to HIHVT at 3 h (*p* <0.05) (Fig 3C). Indeed, PGC-1α protein levels were higher for HIHVT than for SIT at 3 h (*p* < 0.05). SIRT1 mRNA levels remained unchanged throughout the time period investigated (Fig 3D).

**Fig 4:**
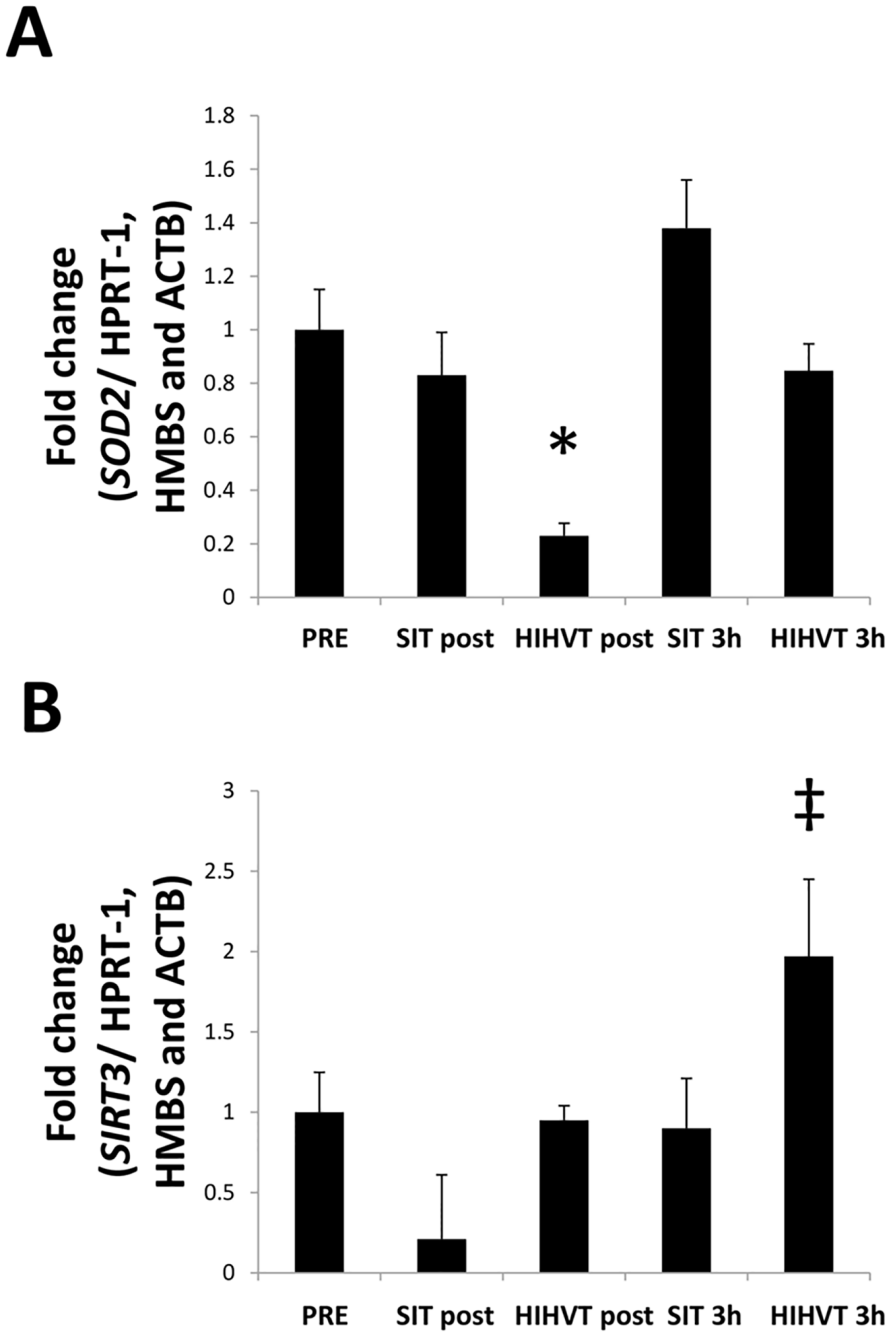
SIRT3 and SOD2 transcription. The SIRT3 mRNA levels were unchanged at all the time points and swimming intensities examined (A). SOD2 mRNA levels decreased at post in response to HIHVT (B). Results are shown as mean ± SEM. * p < 0.05 *vs*. pre.

The mRNA levels of the SIRT3-SOD2 axis were analyzed. SIRT3 (Fig 4A) showed a higher transcriptional response at 3 h in response to HIHVT (*p* < 0.05) if compared to SIT. This effect was found despite SIRT3 did not significantly change if compared to pre. Indeed, there was a tendency toward down-regulation post SIT (*p* = 0.14). In addition, SOD2 mRNA levels (Fig 4B) were unaltered in response to SIT, but decreased significantly at post after HIHVT (*p* < 0.01).

### Downstream PGC-1α-related transcripts

**Fig 5:**
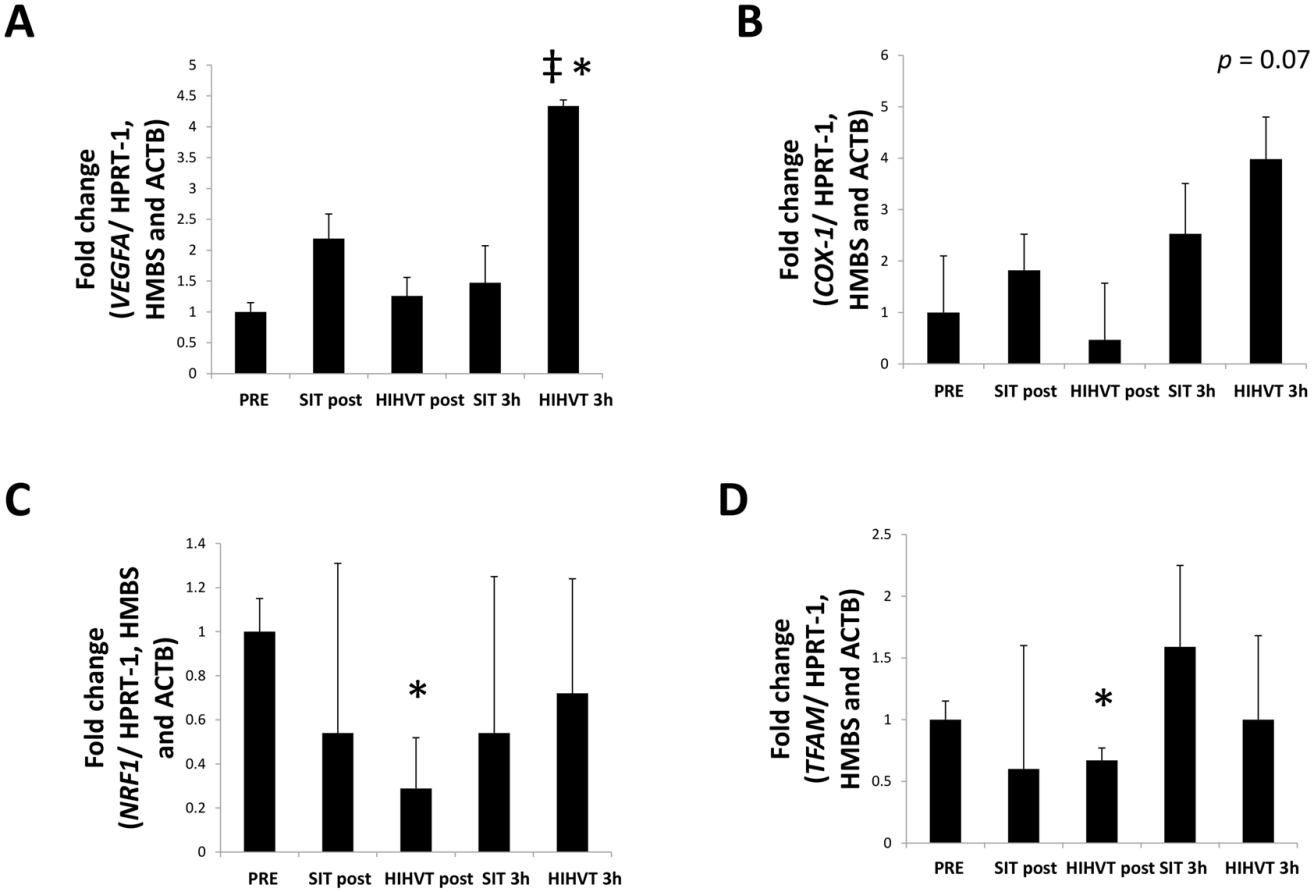
PGC-1α downstream transcripts. HIHVT induced a greater transcription of VEGF than SIT after 3 h (A). HIHVT tended to increase COX-I after 3 h (B). NRF1 (C) and TFAM (D) decreased post HIHVT. Results are shown as mean ± SEM. * *p* < 0.05 *vs*. pre. ‡ *p* < 0.05 *vs*. SIT.

In order to evaluate whether HIHVT-induced PGC-1α expression led to oxidative adaptations, we measured some PGC-1α-related transcripts. VEGF (Fig 5A) remained unchanged in response to SIT. However, HIHVT induced a robust transcription of VEGF at 3 h (*p* < 0.001). Notably, VEGF transcription was higher for HIHVT than for SIT at 3 h (*p* < 0.05). When analyzing several markers of mitochondrial biogenesis, we found a strong trend (*p* = 0.07) towards upregulated COX-1 3 h after HIHVT (Fig 5B). No significant differences were found for NRF1 (Fig 5C) or TFAM (Fig 5D); indeed, both decreased post HIT (*p* < 0.05).

## Discussion

In the present study, we used a randomized crossover design to study molecular signaling in *m*. *triceps brachii*. We described the acute responses of human *m*. *triceps brachii* to HIHVT and SIT swimming. The SIT protocol lasted 36 min where the swimmers performed a volume of 500 m “all out” swimming at a work to rest ratio of 1:8. The HIHVT protocol lasted ~31 min, where the swimmers performed a volume of 2000 m at a work to rest ratio of ~3.5:1. The primary findings of the present study are that HIHVT causes less myocellular membrane damage and similar systemic oxidative damage than SIT. Moreover, high intensity swimming at a constant speed induces more robust AMPK phosphorylation and PGC-1α protein levels at 3 h after exercise.

### Myocellular membrane damage and systemic oxidation

The observation of higher LDH and CK levels after SIT compared to HIHVT suggest greater myocellular membrane rupture after sprint swimming [25]. This effect appears likely to be of muscular origin, although a contribution from other tissues cannot be ruled out. The observation of higher blood lactate concentrations and RPE may suggest a higher internal load in response to SIT compared to HIHVT [26]. With regards to this, it should be highlighted that although both protocols were of similar duration, we did not match them for the same workload. Nevertheless, our results are in accordance with previous findings showing that systemic redox homeostasis was unaffected by swimming at a variety of intensities [27–29], suggesting that well-trained swimmers are highly efficient in managing systemic oxidation processes. Taken together, these observations indicate that SIT is more structurally challenging and might involve anaerobic metabolism to a greater extent than HIHVT.

### Transcriptional antioxidant response within *m*. *triceps brachii*

As illustrated by the lack of systemic redox alterations and of upregulation of SOD2 activation, our results might suggest that swimming does not induce significant oxidative stress in *m*. *triceps brachii*. However, a recent study by Larsen et al. (3) showed that two weeks of arm SIT training hampered mitochondrial adaptations within *m*. *triceps brachii*, attributed to excessive ROS production. However, they performed the analysis on isolated mitochondria. Thus, other ROS production sites in contracting muscle were not taken into account, such as NADPH oxidase (NOX), which is actually considered the main source of ROS within contracting muscle [30]. Since SOD2 mRNA expression in response to exercise [31] seems to be dependent to NOX isoform 2 activity [32], then our results may suggest that neither HIHVT nor SIT are able to evoke sufficient oxidative bursts to induce SOD2 gene expression.

Apart from *m*. *triceps brachii*, the upper body is formed of many muscle groups, such as the biceps, deltoids, dorsalis, and pectoralis, which are compromised during swimming [5]. Over years of exercise, bouts of exercise inducing ROS production may increase the protein level of SOD2, not only in *m*. *triceps brachii* but also in all the muscles involved. In this scenario, the transcriptional response to acute exercise might be blunted due to a previously increased protein content [33]. This consequence would also explain the null transcriptional changes shown for SIRT3. Furthermore, a highly developed antioxidant machinery may explain the different responses shown by PGC-1α and by the SIRT3-SOD2 axis. Indeed, SIRT3 and PGC-1α regulate similar mitochondrial pathways [34,35]. Thus, PGC-1α expression may exhibit an overlapping effect on the mitochondria over SIRT3. It should be noted that SIT tended to reduce SIRT3 mRNA levels if compared to basal levels (Fig 4). Then, we believe that the higher SIRT3 mRNA levels found in response to HIHVT, if compared to SIT but not if compared to basal levels, may be due to the observation that SIT may decrease SIRT3 transcription.

The functional effects of SIRT3 are yet to be elucidated *in vivo* under physiological conditions. Our results and those previously published by others [19,36] suggest that SIRT3 regulation through exercise is a complex process. Indeed, and as previously suggested [19], the stability of SIRT3 may be of key importance and cumulative training bouts could be necessary to show an increase in mRNA. On the other hand, endurance exercise seems to exert a greater stimulus in older subjects than in young subjects regarding the SIRT3-SOD2 pathway [37]. In fact, age reduces SIRT3 content, resulting in an increase of greater magnitude in SIRT3 protein expression in response to endurance exercise than in young subjects [38]. Taken together, the data show that swimming does not induce SOD2 gene expression within *m*. *triceps brachii*, which may be a consequence of insufficient oxidation bursts. In addition, the relationship between SIRT3 and PGC-1α on the control of mitochondrial metabolism under physiological conditions will require further investigation in young healthy subjects.

### Molecular signaling within *m*. *triceps brachii*

Molecular signaling within the arms in response to exercise is still poorly described. The main results suggest that HIHVT should have a greater impact on oxidative metabolism than SIT, as reflected by PGC-1α data. Moreover, AMPK phosphorylation was induced immediately after SIT, whereas in response to HIHVT AMPK phosphorylation occurred after 3 h of rest.

PGC-1α transcriptional activation appeared in response to low cell energy levels. The energy sensors AMPK and SIRT1 are well-known PGC-1α transcriptional and post-transcriptional regulators [22]. Our results suggest that, in already trained arms, PGC-1α expression might occur primarily through AMPK rather than through SIRT1. Accordingly, PGC-1α regulation through SIRT1 requires AMPK [20,39].

In the present study, HIHVT induced late activation of AMPK thorough phosphorylation at the Thr 172 residue, to a significantly greater extent than SIT after 3 h. It must be acknowledged that we did not match the two exercise protocols for a similar workload. Thus, it is possible that the differences in the metabolic demand between HIHVT and SIT may explain the different AMPK activation pattern. However, within the leg skeletal muscle, and regardless of the exercise intensity, AMPK activation thorough phosphorylation at Thr 172 residue occurs early post exercise (i.e., within the first 30 min of exercise cessation) and the return to the basal level is evident after 2 h of rest [40–43]. A possible explanation for this effect may be the different skeletal muscle phenotypes between the arms and legs. Indeed, upper limb muscles have a greater content of glycolytic (i.e., type IIa and IIx) fibers than the legs [44,45]. Recent findings have shown that exercise bouts of 1.5 min induce greater AMPK phosphorylation at Thr 172 in type II glycolytic fibers than continuous exercise [46]. Moreover, AMPK phosphorylation at Thr 172 in response to moderate-intensity exercise is delayed in type II fibers compared to type I fibers [47]. The higher number of type II fibers in the arms may lengthen AMPK activation and/or may prevent its dephosphorylation. It should be noted that AMPK is formed by heterotrimeric complexes with a catalytic α subunit and regulatory γ and β subunits [48]. Although AMPK phosphorylation at the threonine 172 (Thr172) residue of the α subunit is the main form of AMPK activation in response to exercise [48], the other subunits have important implications for molecular signaling in response to exercise [49, 50]. Then, our results are only related to AMPK activation through phosphorylation at the Thr172 residue of the α subunit.

### PGC-1α expression and downstream transcripts

The observation of an increase in the PGC-1α content in response to HIHVT, but not in response to SIT is important as it may reflect a more robust oxidative adaptations induced by HIHVT [8,9,33]. However, one study performed in sedentary women observed that after 12 weeks of swimming, SIT induces greater oxidative adaptations within the deltoid muscle than moderate-intensity swimming [4]. It is likely that these inconsistencies could be due to the fact that we analyzed *m*. *triceps brachii* in trained subjects and therefore our SIT protocol might not be long enough to increase PGC-1α expression.

Skeletal muscle PGC-1α expression in response to exercise modulates oxidative metabolism by promoting mitochondrial biogenesis as well as angiogenesis [8,10]. Our results show that, 3 h after swimming, HIHVT induced higher transcription of VEGF than SIT. Since VEGF is considered a key angiogenic factor directly regulated by PGC-1α [51], it can be speculated that HIHVT-inducing PGC-1α expression may increase *m*. *triceps brachii* capillarization. On the other hand, PGC-1α can control mitochondrial biogenesis through several nuclear factors such as NRF1 and TFAM [11], which were not upregulated in response to any of the trials. However, in order to make up many complexes of the electron transport chain, coordination between mitochondrial and nuclear encoded proteins is needed. In this regard, we found a strong trend (~four-fold change; *p* = 0.07) towards an increase in COX-1 transcription, which is encoded by the mitochondrial genome. Taken together, the data suggest that HIT seems to induce a greater oxidative response within *m*. *triceps brachii*, mainly by increasing the angiogenic response. However, under our experimental conditions, the mitochondrial genome may play an important role in regulating mitochondrial biogenesis in response to HIHVT.

### Strengths and limitations

A strength of the study is that we analyzed the molecular signaling within a less studied muscle (i.e. m. triceps brachii) during a highly automated movement pattern. On the other hand, the limited sample size constitutes the main limitation of the study. In addition, our results are limited to a trained sample and cannot be extrapolated to other populations.

In summary, swimming is a high-quality methodological approach for the examination of *m*. *triceps brachii*. Our results suggest that HIHVT swimming causes lower mechanical stress than SIT but none of the swimming protocols induced neither systemic oxidative response nor muscular transcription of the SIRT3-SOD2 axis. Additionally, HIHVT promotes lengthened/delayed AMPK activation, together with higher PGC-1α protein levels. Therefore, HIHVT seems to induce a more robust oxidative response than SIT within *m*. *triceps brachii* than SIT.
